# Prevalence and associated factors of catastrophic health expenditure among adults with chronic pain in an urban slum of Delhi: a community-based cross-sectional study

**DOI:** 10.3389/frhs.2026.1933208

**Published:** 2026-10-02

**Authors:** Shivani Rao, Kinshuk Gupta, Priyanka Aggarwal, Amod Laxmikant Borle, Nandini Sharma, Mongjam Meghachandra Singh

**Affiliations:** 1 Department of Community Medicine, Maulana Azad Medical College, Delhi, India; 2 Independent Researcher, New Delhi, India

**Keywords:** catastrophic health expenditure, chronic pain, financial hardship, healthcare utilisation, India, out-of-pocket expenditure

## Abstract

**Background:**

Chronic pain is a major and growing public health challenge with a disproportionate burden across sex, age, and socioeconomic groups. As its prevalence continues to rise, it imposes substantial financial hardship on affected households, particularly in low- and middle-income countries (LMICs) where healthcare is largely financed through out-of-pocket expenditure (OOPE). However, the risk of catastrophic health expenditure (CHE) among individuals with chronic pain remains poorly characterised. This study aimed to estimate the prevalence of CHE and identify its associated factors among community-dwelling adults with chronic pain in an urban slum of Delhi.

**Methods:**

A community-based cross-sectional analytical study was conducted among 151 adults with chronic pain (pain persisting >3 months on most or all days) residing in Gokalpuri, North-East Delhi. Participants were recruited by simple random sampling from a household register. CHE was defined as OOPE exceeding 10% of annual household income. Sociodemographic, clinical, and healthcare utilisation variables were assessed using a semi-structured questionnaire. Multivariable logistic regression was used to explore factors associated with CHE.

**Results:**

The prevalence of catastrophic health expenditure was 28.5% (95% CI: 21.4–36.4), with an overshoot of 5.3 percentage points and a mean positive overshoot of 18.4 percentage points (95% CI: 11.6–25.3). Nearly all participants (96.0%) incurred some out-of-pocket expenditure (OOPE), with a median annual direct expenditure of ₹6,000 (IQR: ₹2,400–24,000). Direct medical costs constituted 89.5% of total direct OOPE, with medications being the single largest component. Among the 16 working participants, the median number of workdays lost was 72 days per year (IQR: 60–132), with a range of 24–240 days, corresponding to a mean of 100.5 workdays per participant per year (SD: 58.6). Nearly three-quarters (72.2%) delayed treatment due to financial reasons. Borrowing from family members or relatives was the most common coping strategy to meet treatment expenses (80.1%). On multivariable analysis, daily pain (aOR 7.30; 95% CI: 2.73–19.55) and lower socioeconomic status (aOR 0.05; 95% CI: 0.005–0.41), were significantly associated with CHE, while married status was associated with lower odds of CHE (aOR 0.24; 95% CI: 0.08–0.73).

**Conclusions:**

More than one in four adults with chronic pain in this urban community experienced catastrophic health expenditure. Out-of-pocket spending was dominated by medication costs. Daily pain, lower socioeconomic status, and unmarried/separated/widowed status were associated with greater financial vulnerability. Strengthening public sector pain management capacity, expanding essential medicines availability, and extending social protection schemes may help to reduce the financial hardship chronic pain imposes on India's most vulnerable households.

## 1 Introduction

Chronic pain, defined as pain persisting or recurring for more than three months, is a prevalent and debilitating condition that adversely affects physical, social, and occupational well-being (1). Global prevalence estimates range from 8.7% to 64.4%, with a pooled prevalence of 31%, while estimates from India vary between 19.2% and 62% (2–5). As India undergoes an epidemiological transition characterised by a growing burden of non-communicable diseases, persistent infectious diseases, and injuries, chronic pain is likely to emerge as an even greater public health challenge, with substantial health and economic consequences (6). Barnett et al. found that chronic pain is one of the commonest morbidities to co-occur with other long-term conditions (7). Chronic pain is associated with substantial psychosocial burden, including depression, anxiety, emotional distress, impaired social functioning, and reduced quality of life (8, 9). Many individuals live with chronic pain for years, suffering to such an extreme that they would rather die than continue to live with it (10).

Beyond its impact on health, persistent pain impairs productivity, restricts daily functioning, and increases healthcare utilisation. It imposes a substantial economic burden on individuals, households, and already constrained healthcare systems. Unlike acute, self-limiting illness episodes, chronic pain, by the virtue of its recurrent nature and long-term care, necessitates sustained healthcare utilisation: repeated consultations, ongoing medication, periodic investigations, and in some cases physiotherapy or rehabilitative care over months to years, generating a cumulative financial burden that a single episode cost-of-illness study might not account for (1, 11).

Studies have reported that patients living with chronic pain incur more than twice the annual expenditure of individuals without pain, largely due to increased healthcare utilisation, productivity losses, and absence from work (11). In fact, the economic impact incurred due to chronic pain exceeds that of many other health conditions because of its effects on absenteeism, presenteeism, reduced productivity, and premature exit from the labour market (12). In the United States, lost productive time due to common pain conditions has been estimated at 61.2 billion dollars annually (13). Studies suggest that individuals with chronic pain are substantially more likely to leave employment compared to those without pain. A community-based study from Gadchiroli, India, reported that adults with chronic pain incurred considerable expenditure on medical consultations and medications, while also losing more than 24,000 work-days annually (14). McQuay, therefore, argues that the imperative to improve the management of chronic pain is grounded not only in humanitarian considerations but also in economic ones (15).

Catastrophic health expenditure (CHE) is defined as out-of-pocket expenditure exceeding 10% or 25% of total household consumption or income, or exceeding 40% of household capacity-to-pay after accounting for basic subsistence needs (16). It acts as a proxy indicator of the magnitude of out-of-pocket health spending that places substantial pressure on household resources and compromises a household's ability to meet other basic needs. It can force households to reduce spending on other essential goods and services, draw on savings, borrow money, or sell assets, thereby exposing them to financial hardship and economic vulnerability and increasing reliance of social welfare schemes.

India has a pluralistic health system in which publicly funded primary, secondary, and tertiary care operates alongside a large private healthcare sector. Public-sector services are organised through a tiered referral system, while healthcare delivery and financing remain substantially fragmented across public and private providers. As health systems increasingly face pressures to allocate resources efficiently, understanding the economic implications of chronic pain has become a public health priority (12, 17, 18). Urban slum residents may be particularly vulnerable to chronic pain because of physically demanding occupations, poor living and working conditions, and limited access to preventive and healthcare services (19, 20). At the same time, limited and erratic financial resources may make recurrent healthcare costs difficult to absorb. The existing Indian CHE evidence has focused on either non-communicable diseases (21), specific chronic condition (22) or vulnerable population groups like the elderly (23), rather than on chronic pain as a condition in its own right within a community-based sample. To the best of our knowledge, this is among the first community-based studies in India to estimate the prevalence and factors associated with catastrophic health expenditure among adults living with chronic pain in an urban slum of Delhi.

## 2 Materials and methods

### 2.1 Study design and setting

This was a community-based cross-sectional analytical study conducted in the urban field practice area of Gokalpuri, North-East Delhi, India. Gokalpuri is a densely populated, lower-middle-income urban locality representative of peri-urban Delhi. The area is served by the Urban Health Training Centre of the Department of Community Medicine and maintains a household register that formed the sampling frame for this study. The study area is characterised by mixed residential and commercial zones, with a predominantly lower-middle-income population engaged in informal and small-scale employment.

### 2.2 Study population

Participants were eligible if they were (i) permanent residents of the Gokalpuri field practice area for at least six months, (ii) aged 18 years or above, (iii) willing to provide informed consent. Chronic pain was defined using the Graded Chronic Pain Scale–Revised (GCPS-R) (24) as pain persisting for more than three months and occurring on most or all days. Participants requiring healthcare were advised to seek appropriate care at the Urban Health Training Centre. Individuals who were unable to understand the questionnaire adequately were excluded from the study.

### 2.3 Sample size and sampling

The sample size was calculated using the single population proportion formula (*n* = Z^2^pq/d^2^), assuming a prevalence of catastrophic health expenditure of 25.9% among adults with severe pain, based on a previous study from South Korea. Using a 95% confidence level and an absolute precision of 7%, the minimum required sample size was estimated to be 151 participants (25). Participants were recruited using simple random sampling. A sampling frame was constructed from the household register of the urban field practice area maintained by the Department of Community Medicine. Households with at least one eligible participant were identified through a preliminary household survey. A random number sequence was generated using a computer-based random number generator, and households were selected in the order of their assigned random numbers. Where more than one eligible participant was present in a household, one participant was selected using the Kish grid method to avoid clustering of multiple participants from the same household.

All field investigators underwent a structured training programme conducted by the principal investigator prior to commencement of data collection. Training covered: (i) detailed familiarisation with the study questionnaire and interview procedures, including probing techniques for chronic pain, healthcare expenditure and healthcare utilisation; (ii) standardised anthropometric measurement procedures with inter-rater reliability testing; (iii) informed consent procedures; and (iv) data recording and quality assurance.

### 2.4 Outcome variable

Catastrophic health expenditure (CHE) was assessed using a semi-structured questionnaire administered in Hindi by trained field investigators. The study tool was pilot tested among 20 participants prior to field implementation to assess its clarity, comprehensibility, and feasibility. Based on the feedback obtained during the pilot phase, necessary modifications were made before its final deployment in the field.

Catastrophic health expenditure (CHE) was defined as out-of-pocket healthcare expenditure exceeding 10% of annual household income (26). Let $OOP_{i}$ denote annual out-of-pocket healthcare expenditure for household *i*, $Y_{i}$ denote annual household income, and z=0.10 denote the CHE threshold. CHE was defined as:

$$CHE_{i}=\{\begin{array}{cc} 1, & \mathrm{if}\;\frac{OOP_{i}}{Y_{i}}>0.10\\ 0, & \mathrm{otherwise} \end{array}$$

Overshoot was calculated as the mean excess of the OOP expenditure-to-income ratio above the CHE threshold across all participants:

$$O=\frac{1}{N}\overset{N}{\underset{i=1}{\sum }}\;\max\left(0,\frac{OOP_{i}}{Y_{i}}-z\right)$$

where *N* denotes the total number of participants. Participants whose OOP expenditure did not exceed 10% of annual household income were assigned an overshoot of zero.

Mean positive overshoot (MPO) was calculated as the mean excess above the CHE threshold among participants experiencing CHE:

$$MPO=\frac{\sum_{i=1}^{N}\;\max\left(0,\frac{OOP_{i}}{Y_{i}}-z\right)}{\sum_{i=1}^{N}\;I\left(\frac{OOP_{i}}{Y_{i}}>z\right)}$$

where I(⋅) is an indicator function taking a value of 1 when OOP expenditure exceeds the CHE threshold and 0 otherwise.

Information on annual household income and out-of-pocket healthcare expenditure related to chronic pain treatment was collected. Annual household income was calculated by aggregating the annualised income of all earning household members. For members with regular monthly income, the reported monthly income was multiplied by 12. For individuals engaged in informal or unorganised employment, the average daily wage and number of days worked during the preceding month were recorded. Monthly earnings were calculated from these values and annualised to estimate annual income. Income from all earning members was then summed to obtain the total annual household income.

The out-of-pocket expenditure of chronic pain was assessed in terms of direct medical costs, direct non-medical costs, and indirect costs. Direct medical costs included out-of-pocket expenditure on medications, doctor consultations, diagnostic investigations, hospital fees, and physiotherapy in a year. Direct non-medical costs comprised annual expenditure on transportation, food, and lodging incurred while seeking healthcare. Participants were asked to recall expenditure separately for each of these cost components, and component-wise values were summed to derive total direct out-of-pocket expenditure. Indirect costs were assessed as productivity losses due to chronic pain and measured by the number of workdays lost during the preceding year.

### 2.5 Predictor variables

Information on sociodemographic characteristics including age, gender, marital status, family type, educational attainment, occupation, and socioeconomic status was obtained using a semi-structured questionnaire. Socioeconomic status was assessed using the BG Prasad Socioeconomic Scale (2026) (27).

Clinical characteristics included the frequency of chronic pain, multisite pain involvement, and functional impairment. Participants were classified as experiencing pain on most days or all days during the preceding three months. Multisite pain was defined as pain involving more than one anatomical site. Functional impairment was assessed based on self-reported limitations in everyday activities such as walking, self-care, household work, and work outside the home attributable to pain during the previous month.

Information on healthcare utilisation included the type of healthcare provider and healthcare facility consulted, the number of doctors consulted for pain, and polypharmacy (use of five or more medications per day.) (28)

Economic variables encompassed financial autonomy, healthcare decision-making autonomy, perceived affordability of treatment, financial constraints related to treatment, cost-related care seeking delay, and health insurance status.

Anthropometric measurements were obtained using standard procedures. Weight was measured using a calibrated digital weighing scale and height using a portable stadiometer. Body mass index was calculated as weight in kilograms divided by height in metres squared (kg/m^2^) and classified according to the World Health Organization criteria (29).

### 2.6 Statistical analysis

All data were collected electronically using KoBoCollect Toolbox (KoBoToolbox, Harvard Humanitarian Initiative, USA), exported into a structured spreadsheet, cleaned for inconsistencies and range errors, and analysed using Stata version 19 BE (StataCorp LLC, College Station, TX, USA). Continuous variables were assessed for normality using the Shapiro–Wilk test. Normally distributed variables are reported as mean ± standard deviation (SD), while non-normally distributed variables are presented as median (interquartile range, IQR). Categorical variables are reported as frequency and percentage.

Associations between categorical predictors and catastrophic health expenditure were assessed using the Chi-square test or Fisher's exact test as appropriate. A parsimonious multivariable model was then specified using a smaller set of variables selected *a priori* on clinical and public health relevance. These variables were entered simultaneously into a multivariable binary logistic regression model using a forced-entry (enter) approach. All eligible variables entered together in one block, without stepwise selection, to avoid optimistic overfitting and to obtain mutually adjusted estimates. Results are reported as adjusted odds ratios (aOR) with 95% confidence intervals and two-tailed *p*-values. Model fit was assessed using the Hosmer-Lemeshow goodness-of-fit test, while multicollinearity was assessed using variance inflation factors (VIF). A two-tailed significance threshold of *α*=0.05 was applied throughout for all primary analyses.

### 2.7 Ethical approval

The study was conducted in accordance with the ethical principles laid down in the Declaration of Helsinki (2013 revision). Ethical approval was obtained from the Institutional Ethics Committee of the parent institution prior to commencement of fieldwork (IEC/MAMC/116/04/2025/439). Written informed consent was obtained from all participants after explaining the study objectives, procedures, voluntary nature of participation, confidentiality safeguards, and the right to withdraw at any stage without consequence. All data were anonymised prior to analysis, and no personally identifiable information was included in the final dataset.

## 3 Results

### 3.1 Sociodemographic profile

Table 1 presents the sociodemographic characteristics of the study participants. The mean age of the participants was 53.0 years (53 ± 10.8), with nearly two-thirds (65.6%) aged 50 years or older. Most participants were women (74.2%), homemakers (68.9%), married (84.1%), belonged to nuclear families (72.8%), and practised Hinduism (97.4%). Primary education was the most common educational attainment (34.4%), while 27.2% of participants were illiterate. Nearly half of the participants belonged to the upper-middle socioeconomic class (45.7%), and 44.4% were overweight.

**Table 1:** Sociodemographic characteristics of study participants (*N* = 151).

Variable	n (%)
Age Classification	
<40 years	15 (9.9)
40–49 years	37 (24.5)
50–59 years	49 (32.5)
60–69 years	37 (24.5)
≥70 years	13 (8.6)
Gender	
Female	112 (74.2)
Male	39 (25.8)
Occupation	
Homemaker	104 (68.9)
Retired	31 (20.5)
Working	16 (10.6)
Marital Status	
Married	127 (84.1)
Divorced/Widowed/Separated	21 (13.9)
Single	3 (2.0)
Family Type	
Nuclear family	110 (72.8)
Extended family	24 (15.9)
Joint family	17 (11.3)
Educational Status	
Illiterate	41 (27.2)
Primary education	52 (34.4)
Middle school	15 (9.9)
High school	25 (16.6)
Diploma/Intermediate	13 (8.6)
Graduate	4 (2.7)
Postgraduate	1 (0.7)
Socioeconomic Status	
Lower class	8 (5.3)
Lower middle class	24 (15.9)
Middle class	45 (29.8)
Upper middle class	69 (45.7)
Upper class	5 (3.3)
Religion	
Hindu	147 (97.4)
Muslim	1 (0.7)
Other religion	3 (2.0)
Body Mass Index	
Underweight	7 (4.6)
Normal	51 (33.8)
Overweight	67 (44.4)
Obese	26 (17.2)

### 3.2 Economic burden

#### 3.2.1 Out-of-pocket health expenditure

Nearly all participants (96.0%) incurred some out-of-pocket (OOP) expenditure during the reference period. Direct medical costs accounted for 89.5% of total OOP expenditure, while direct non-medical costs contributed the remaining 10.5%. The median annual direct out-of-pocket expenditure related to chronic pain was ₹6,000 (IQR: ₹2,400–24,000) (Table 2).

**Table 2:** Direct medical and non-medical expenditure among participants.

Expenditure Category	Mean ± SD (₹)	Median (IQR), ₹	Participants with Expenditure, n (%)
Total out-of-pocket expenditure	17,623.3 ± 28,514.8	₹6,000 (₹2,400 to ₹24,000)	145 (96.0)
Direct medical expenditure	15,767.7 ± 26,571.0	₹6,000 (₹2,400 to ₹18,000)	144 (95.4)
Direct non-medical expenditure	1,855.6 ± 5,339.8	₹0 (₹0 to ₹1,200)	50 (33.1)

Medications constituted the largest component of out-of-pocket expenditure, accounting for more than two-thirds of the total expenditure, followed by doctor consultations, transportation, and diagnostic investigations. Expenditure on physiotherapy, food, hospital fees, and lodging contributed relatively smaller proportions to the overall direct out-of-pocket expenditure. Percentages are expressed as a proportion of total direct out-of-pocket expenditure across the full study sample (Figure 1).

**Figure 1:**
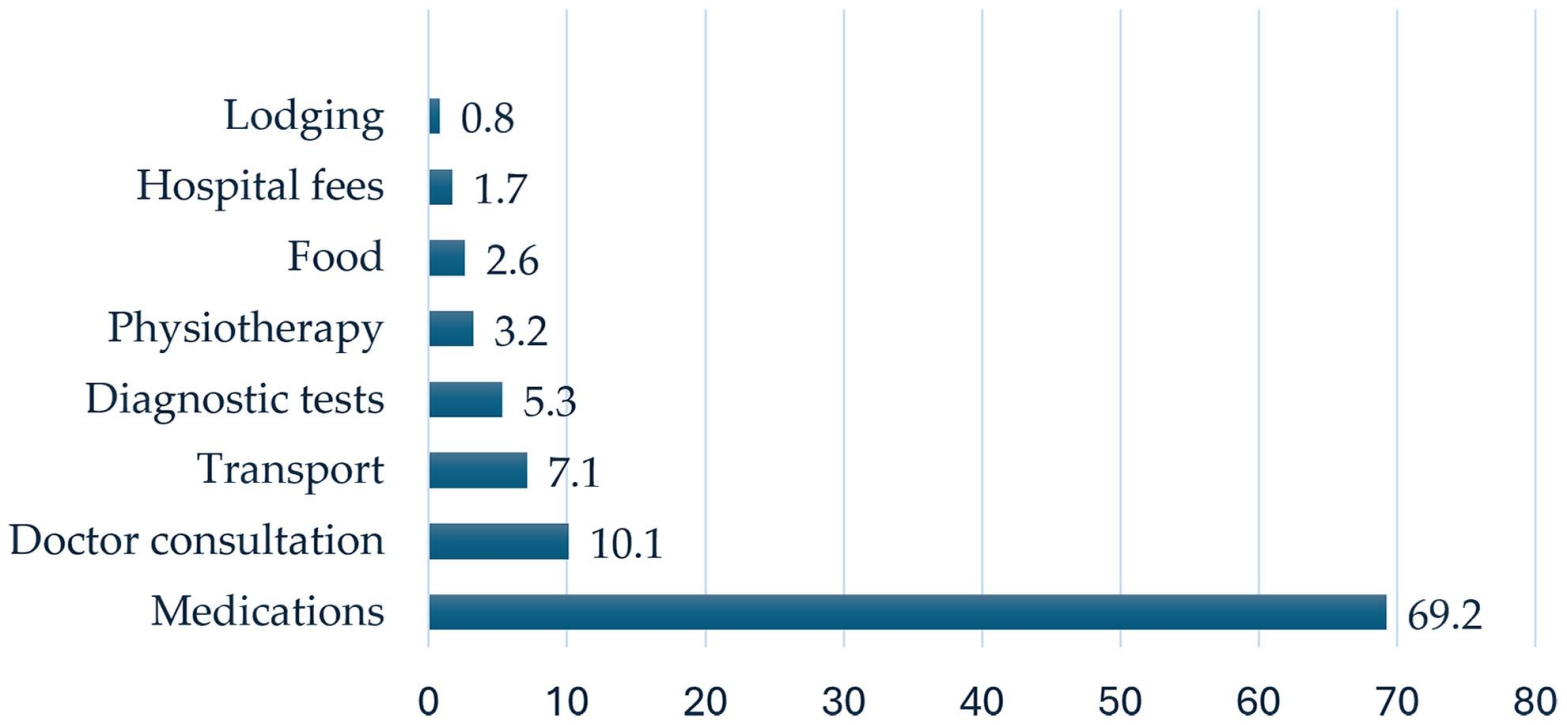
Distribution of annual out-of-pocket health expenditure by cost component.

#### 3.2.2 Workdays lost attributable to chronic pain

Among the 16 working participants, the median number of workdays lost was 72 days per year (IQR: 60–132), with a range of 24–240 days. It resulted in a total of 1,608 workdays lost over one year, corresponding to a mean of 100.5 workdays per participant per year (SD: 58.6).

#### 3.2.3 Catastrophic health expenditure

The prevalence of catastrophic health expenditure was 28.5% (95% CI: 21.4–36.4) with an overshoot of 5.3 percentage points. Among participants experiencing CHE, the mean positive overshoot, was 18.4 percentage points (95% CI: 11.6–25.3). Figure 2 presents the prevalence of catastrophic health expenditure at various thresholds (*N* = 151).

**Figure 2:**
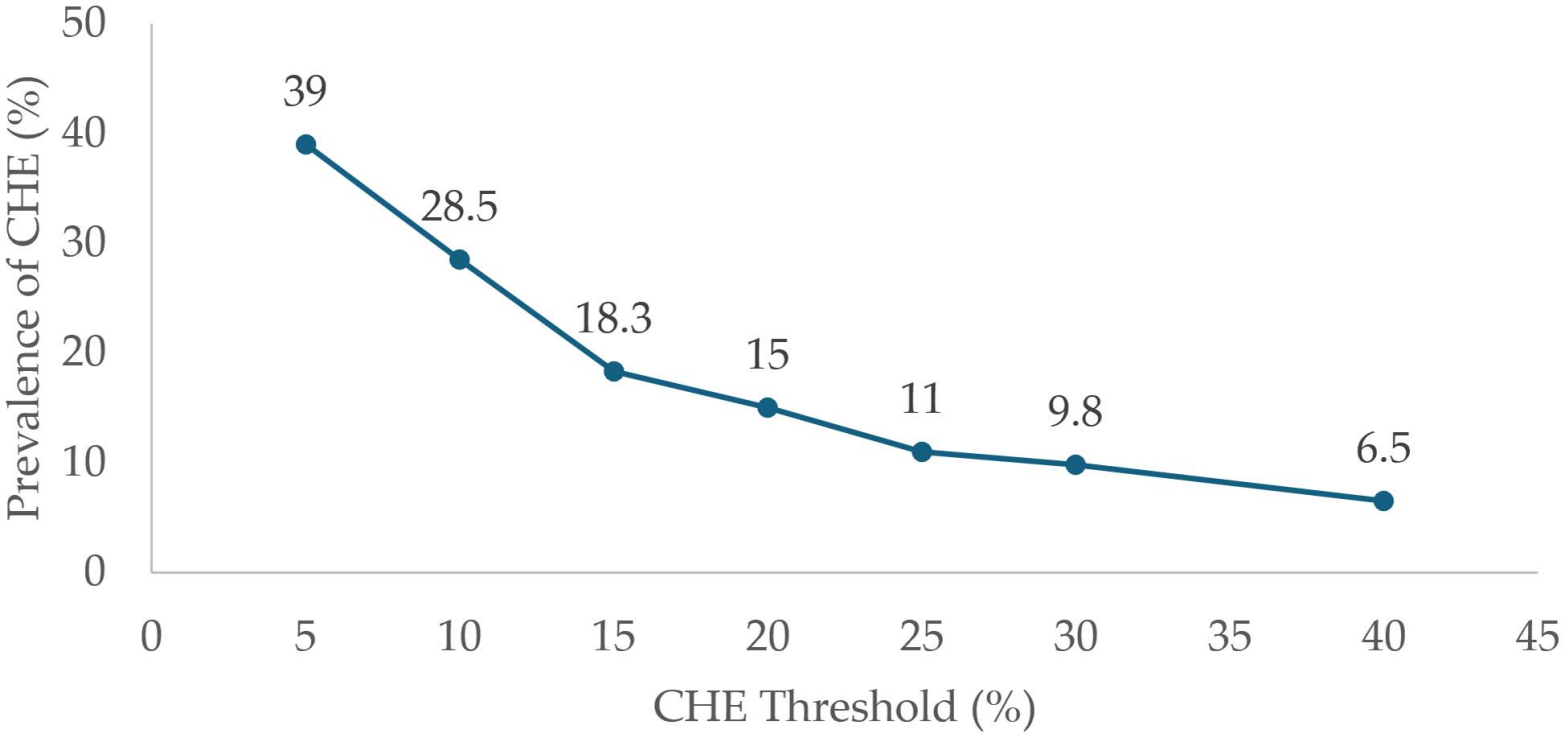
Prevalence of catastrophic health expenditure across various thresholds.

Participants experiencing catastrophic health expenditure incurred substantially greater annual healthcare costs than those without catastrophic expenditure (Table 3). The median annual out-of-pocket expenditure among participants with CHE was ₹36,000 (IQR: ₹21,000 to ₹55,200), which was eight times higher than that among participants without CHE (₹4,500; IQR: ₹2,400 to ₹8,400; *p* < 0.001). Similarly, median annual direct medical expenditure was significantly higher in the CHE group (₹30,000; IQR: ₹18,000 to ₹48,000) compared with the non-CHE group (₹3,600; IQR: ₹2,400 to ₹8,400; *p* < 0.001). Direct non-medical expenditure was also significantly greater among participants with CHE, with a median annual expenditure of ₹1,200 (IQR: ₹0 to ₹6,600), whereas participants without CHE reported no non-medical expenditure at both the median and interquartile range (₹0; IQR: ₹0 to ₹0; *p* < 0.001).

**Table 3:** Comparison of out-of-pocket expenditure between participants with and without catastrophic health expenditure (*N* = 151).

Expenditure Category (Annual)	No CHE (*n* = 108)	CHE (*n* = 43)	*p*-value
Total out-of-pocket expenditure	₹4,500 (₹2,400 to ₹8,400)	₹36,000 (₹21,000 to ₹55,200)	<0.001
Direct medical expenditure	₹3,600 (₹2,400 to ₹8,400)	₹30,000 (₹18,000 to ₹48,000)	<0.001
Direct non-medical expenditure	₹0 (₹0 to ₹0)	₹1,200 (₹0 to ₹6,600)	<0.001

*P*-values were obtained using the Mann–Whitney U test because expenditure data were not normally distributed.

#### 3.2.4 Financial autonomy, treatment-related decision-making and coping mechanism

Participants demonstrated considerable financial vulnerability in accessing healthcare. More than two-thirds (67.3%) did not have their own financial resources to meet healthcare expenses. Healthcare decision-making was predominantly shared within households, with nearly half (48.3%) reporting joint decision-making with their spouse or partner, while 30.5% made healthcare decisions independently and 21.2% relied on their family to make such decisions.

The financial impact of chronic pain was substantial. Nearly half of the participants (48.3%) experienced financial constraints related to treatment, and almost three-quarters (72.2%) reported delaying healthcare because of financial reasons. Only 7.3% considered treatment costs to be fully affordable, whereas 69.5% perceived them as partially affordable and 23.2% as unaffordable. To meet treatment-related expenses, most participants relied on informal financial support, primarily borrowing money from family members or relatives (80.1%), followed by the use of personal savings or salary (39.1%). Financial protection through health insurance was uncommon, with only 6.0% of participants reporting any insurance coverage.

### 3.3 Bivariate analysis

The prevalence of catastrophic health expenditure was significantly higher among participants aged ≥50 years than among those aged <50 years (35.4% vs. 15.4%; *p* = 0.010). Higher proportions of CHE were also observed among participants who were not currently married as compared to currently married individuals (62.5% vs. 22.0%; *p* < 0.001), among those without formal education compared with those having any formal education (48.8% vs. 20.9%; *p* = 0.001), and among participants belonging to lower socioeconomic strata compared with higher socioeconomic groups (65.6% vs. 18.5%; *p* < 0.001). Although CHE was more frequent among participants from non-nuclear families than those from nuclear families (39.0% vs. 24.5%), the association did not reach statistical significance (*p* = 0.080). (Table 4).

**Table 4:** Factors associated with catastrophic health expenditure among participants with chronic pain (*N*=151).

Variable	Category	N	CHE n (%)	No CHE (%)	*p*-value
Sociodemographic domain					
Gender	Female	112	33 (29.5%)	79 (70.5%)	0.649
	Male	39	10 (25.6%)	29 (74.4%)	
Age group	<50 years	52	8 (15.4%)	44 (84.6%)	0.010 *
	≥50 years	99	35 (35.4%)	64 (64.6%)	
Occupation	Homemaker	104	31 (29.8%)	73 (70.2%)	0.590
	Retired/Working	47	12 (25.5%)	35 (74.5%)	
Marital status	Not currently married	24	15 (62.5%)	9 (37.5%)	<0.001 *
	Married	127	28 (22.0%)	99 (78.0%)	
Family type	Nuclear family	110	27 (24.5%)	83 (75.5%)	0.080
	Non-nuclear family	41	16 (39.0%)	25 (61.0%)	
Educational status	Illiterate	41	20 (48.8%)	21 (51.2%)	0.001 *
	Any formal education	110	23 (20.9%)	87 (79.1%)	
Socioeconomic status	Lower & lower-middle class	32	21 (65.6%)	11 (34.4%)	<0.001 *
	Middle, upper-middle & upper class	119	22 (18.5%)	97 (81.5%)	
Religion	Hindu	147	43 (29.3%)	104 (70.7%)	0.578
	Minority religion	4	0 (0.0%)	4 (100.0%)	
BMI category	Underweight/Normal	58	15 (25.9%)	43 (74.1%)	0.574
	Overweight/Obese	93	28 (30.1%)	65 (69.9%)	
Clinical domain					
Frequency of pain	Frequent pain	122	24 (19.7%)	98 (80.3%)	<0.001 *
	Daily pain	29	19 (65.5%)	10 (34.5%)	
Multisite pain	No	127	34 (26.8%)	93 (73.2%)	0.286
	Yes	24	9 (37.5%)	15 (62.5%)	
Functional impairment	Mild impairment	26	10 (38.5%)	16 (61.5%)	0.462
	Moderate impairment	84	22 (26.2%)	62 (73.8%)	
	Severe impairment	41	11 (26.8%)	30 (73.2%)	
Healthcare utilisation domain					
Specialist consultation	Non-specialist	128	25 (19.5%)	103 (80.5%)	<0.001 *
	Specialist	23	18 (78.3%)	5 (21.7%)	
Healthcare facility utilized	Non Private	82	15 (18.3%)	67 (81.7%)	0.003 *
	Private facility	69	28 (40.6%)	41 (59.4%)	
Number of doctors consulted	0–1 doctor	94	20 (21.3%)	74 (78.7%)	0.012 *
	≥2 doctors	57	23 (40.4%)	34 (59.6%)	
Polypharmacy	<5 tablets/day	125	26 (20.8%)	99 (79.2%)	<0.001 *
	≥5 tablets/day	26	17 (65.4%)	9 (34.6%)	

*N*=151. CHE, Catastrophic Health Expenditure. Chi-square test was used for all comparisons except Religion, for which Fisher's exact test was used owing to small expected cell counts. * Statistically significant at *p*<0.05.

In the clinical domain, participants reporting daily pain had a significantly higher prevalence of CHE than those experiencing pain on most days (65.5% vs. 19.7%; *p* < 0.001). With respect to healthcare utilisation, CHE was significantly more common among participants consulting specialists (78.3% vs. 19.5%; *p* < 0.001), utilising private healthcare facilities (40.6% vs. 18.3%; *p* = 0.003), consulting two or more doctors (40.4% vs. 21.3%; *p* = 0.012), and reporting polypharmacy (65.4% vs. 20.8%; *p* < 0.001).

### 3.4 Multivariable binary logistic regression

In the multivariable model, daily pain (versus pain on most days) was associated with substantially higher odds of catastrophic health expenditure (aOR=7.30; 95% CI: 2.73–19.55). (Table 5) Participants belonging to a middle or upper socioeconomic class were associated with markedly lower odds than belonging to a lower socioeconomic class (aOR=0.05; 95% CI: 0.005–0.41). Married status, compared with not currently married, was associated with lower odds of CHE (aOR=0.24; 95% CI: 0.08–0.73). Age ≥50 years showed a positive association that did not reach statistical significance after adjustment (aOR=2.28; 95% CI: 0.81–6.40), and gender was not associated with CHE in the adjusted model (aOR=1.04; 95% CI: 0.37–2.94).

**Table 5:** Multivariable binary logistic regression — exploratory factors associated with catastrophic health expenditure among participants with chronic pain (*N* = 151).

Variable	Category	Unadj. OR	95% CI	p	Adj. OR	95% CI	p
Age group	≥50 years vs <50 years	3.01	1.27–7.10	0.012*	2.28	0.81–6.40	0.118
Marital status	Currently married vs Not currently married	0.17	0.07–0.43	<0.001*	0.24	0.08–0.73	0.011*
Gender	Female vs Male	0.83	0.36–1.88	0.649	1.04	0.37–2.94	0.942
Frequency of pain	Daily pain vs Frequent pain	7.76	3.20–18.82	<0.001*	7.30	2.73–19.55	<0.001*
Socioeconomic status	Middle/Upper Middle/Upper vs Lower & Lower-middle	0.12	0.05–0.28	<0.001*	0.05	0.005–0.41	0.006*

Model fit: LR *χ*² = 47.54 | *p* < 0.001 | Cragg and Uhler's R² = 0.387 | Hosmer-Lemeshow *χ*² = 9.27, *p* = 0.099 | Mean VIF = 1.08.

Unadjusted ORs were obtained from univariable logistic regression models. Adjusted ORs were estimated from the multivariable logistic regression model. Reference categories: Age <50 years; Marital status, Not currently married; Gender, Male; Frequency of pain, Frequent pain; Socioeconomic status, Lower and lower-middle. OR, odds ratio; 95% CI, 95% confidence interval. *p<0.05.

## 4 Discussion

In the present community-based cross-sectional study conducted to estimate the prevalence and explore factors associated with CHE among participants living with chronic pain, it was found that 28.5% of participants experienced catastrophic health expenditure. Nearly all participants (96.0%) incurred some out-of-pocket expenditure, with direct medical costs constituting 89.5% of total direct OOPE and medications emerging as the single largest expenditure component. In the multivariable model adjusting for age, marital status, gender, pain frequency, and socioeconomic status, daily pain and lower socioeconomic status were associated with higher odds of CHE, while married status was associated with lower odds.

Our estimates are comparable to national prevalence of 24.9% based on the 71st round (2014 utilisation survey) of NSSO (30). Higher estimates have been reported among individuals with non-communicable diseases (37.2%–65.7%) and among households with chronic illness (21, 31). Globally, CHE among patients with severe pain was found to be 25.9% in South Korea (25). Another systematic review from China by Zhang et al. reported the overall prevalence of CHE to be 25.2% (32). The variation in CHE estimates across studies may be explained by differences in study populations, disease characteristics, operational definitions and variations in health financing and out-of-pocket spending across settings.

The strong association between daily pain and CHE observed in this study is consistent with a dose–response relationship reported in the study by Shin et al. in South Korea, where the prevalence of CHE rose progressively from 3.3% among pain-free individuals to 11.1% among those with pain and 25.9% among those with severe pain, with the adjusted odds of CHE more than doubling between the pain and severe-pain groups (25). This gradient supports the plausibility of our finding that participants with daily pain, reflecting a more persistent and high-impact pain state, face a substantially higher burden of catastrophic expenditure than those with intermittent pain, likely driven by more frequent consultations, higher medication use, and greater cumulative healthcare utilisation over time (11). This is further compounded by the substantially greater number of workdays lost among working participants with chronic pain, which adds an indirect productivity-related burden on top of direct medical costs.

While marital status emerged as a protective factor in the present study, the evidence remains sparse and inconsistent (33). A possible explanation could be that married individuals benefit from pooled household resources and shared financial responsibilities, which may enhance their ability to absorb healthcare costs without experiencing financial catastrophe. In contrast, widowed, separated, or single individuals may have reduced economic security and social support. Choi et al. reported lower odds of chronic pain among widowed older adults relative to their married counterparts, while Wade et al., studying patients already living with chronic pain, found that widowed individuals reported less emotional distress and depression than those who were divorced or separated, suggesting that adaptive coping strategies following major life events may mitigate some of the psychosocial burden of chronic pain even where marital protection against its onset is not observed (34, 35).

The finding of lower socioeconomic status being more prone to CHE is consistent with evidence from India and other LMICs (32, 33, 36–41). NSSO data indicate that the poorest households spend nearly twice the proportion of their consumption expenditure on healthcare compared with the richest households (23). Chronic illnesses such as chronic pain can rapidly deplete household resources, forcing families to rely on borrowing, asset sales, and other distress financing mechanisms to meet healthcare expenses (42). In contrast to wealthier households, poorer families often forgo or delay healthcare to avoid catastrophic expenditure, which may lead to worse health and economic outcomes over time.

The findings of this study have important policy implications. Primary-care physicians and medical officers frequently lack structured training in stepwise pain management. Further, India faces a shortage of pain-medicine specialists relative to population need, with active palliative and pain-care centres covering a density of only about four per ten million population nationally (43). Standardising chronic pain assessment and first-line prescribing protocols at the primary care level could support earlier, more consistent management of pain in the community. Ensuring uninterrupted public-sector availability of essential analgesics consistent with India's National List of Essential Medicine, alongside basic diagnostic support (radiography, routine laboratory investigations), would address the medication-cost burden that constituted the largest single component of out-of-pocket expenditure in this study. Public-sector strengthening should explicitly prioritise accessibility and affordability for economically vulnerable households, including through targeted enrolment support for social protection schemes.

This study has several strengths. To our knowledge, it is among the first community-based studies from India to estimate catastrophic health expenditure among individuals living with chronic pain. Unlike studies focusing on specific pain conditions or hospital-based populations, the present study captured a broad spectrum of chronic pain patients in a community setting, thereby improving the generalizability of the findings. The study also examined a range of sociodemographic, clinical, healthcare utilisation, and economic characteristics in relation to catastrophic expenditure, providing a broad descriptive picture of financial hardship in this population.

However, certain limitations should be acknowledged. The cross-sectional design precludes establishing causal relationships between the variables examined and catastrophic health expenditure. The expenditure data were based on self-report and may be subject to recall bias or social desirability bias. The study was conducted in a single urban field practice area of Delhi, which may limit the generalizability of the findings to other geographic settings. Indirect productivity costs were limited to self-reported workdays lost among participants in paid employment and were not monetised. Caregiver, household productivity, and intangible costs were not monetised, potentially underestimating the overall economic burden. CHE was calculated using expenditure directly attributable to the participant's chronic pain, rather than total household healthcare expenditure, which may have resulted in an underestimation of the overall household financial burden.

## 5 Conclusion

Catastrophic health expenditure is highly prevalent among individuals living with chronic pain, affecting more than one-fourth of the study participants. In the adjusted model, daily pain and lower socioeconomic status were associated with higher odds of catastrophic expenditure, while married status was associated with lower odds. The findings highlight that the financial burden of chronic pain extends beyond the disease itself and is shaped by underlying socioeconomic and social-support inequities. Strengthening chronic pain management within primary healthcare, improving the availability of essential medicines in the public sector, and expanding financial risk protection mechanisms targeted at economically vulnerable households are likely to help reduce out-of-pocket expenditure and protect against financial hardship.

## Data Availability

The raw data supporting the conclusions of this article will be made available by the authors, without undue reservation.
